# Determination of the Molecular Mechanism of Torularhodin against Hepatic Oxidative Damage by Transcriptome Analysis

**DOI:** 10.1155/2019/7417263

**Published:** 2019-07-14

**Authors:** Jiayi Li, Yahui Guo, Yuliang Cheng, Fuwei Pi, Weirong Yao, Yunfei Xie, He Qian

**Affiliations:** Department of School of Food Science and Technology, Jiangnan University, Wuxi 214122, China

## Abstract

Torularhodin, extracted from *Sporidiobolus pararoseus*, is a significant carotenoid that is similar to lycopene in structure. Some studies have indicated torularhodin as having antioxidative activities. However, it has not been thoroughly studied with respect to its antioxidative activity and molecular mechanisms in liver injury. Therefore, the aim of this study was to elucidate the antioxidative activity of torularhodin against hydrogen peroxide- (H_2_O_2_-) induced damage and the mechanism involved through transcriptome analysis and to explore its antioxidant potential. BRL cells were first subjected to H_2_O_2_ damage and then treated with torularhodin. The results showed that at 10^−5^ g/ml, torularhodin had significant protective effects against H_2_O_2_-induced oxidative damage. Morphological and immunofluorescence staining showed that torularhodin could maintain cell integrity and enhance the activity of antioxidant enzymes in the cells. According to transcriptome analysis, 2808 genes were significantly differentially expressed (1334 upregulated and 1474 downregulated) after torularhodin treatment. These genes were involved in three major Gene Ontology categories (biological process, cellular component, and molecular function). Moreover, torularhodin was involved in some cellular pathways, such as cancer inhibition, antioxidation, and aging delay. Our data highlighted the importance of multiple pathways in the antioxidative damage of liver treated with torularhodin and will contribute to get the molecular mechanisms of torularhodin inhibition of hepatic oxidative damage.

## 1. Introduction

The liver is the most important metabolic organ, accounting for approximately 2% of the total body weight. More than 500 significant functions are performed by this organ, such as conversion of food components to critical blood components, storage of vitamins and minerals, manufacture of many vital plasma proteins and minerals, maintenance of hormonal balance and metabolism, and detoxification of toxic wastes in the body [1]. Numerous chemicals have been reported to cause liver injury, such as drugs, pollutants, fried foods, and alcohol [2–5]. Although drugs are the most effective way to treat diseases, most of them are metabolized in the liver and kidneys and can eventually lead to hepatotoxicity. Although alcohol has been stated to be beneficial to health when taken in moderation, it is still harmful to the liver. Alcohol can also increase the metabolic pressure in the liver and cause oxidative damage to the organ, which can modify the structure and function of proteins, damage DNA, and lead to fatty liver and cirrhosis in severe cases [6]. Alcohol consumption has resulted in 3.3 million deaths worldwide, accounting for 5.9% of all deaths in 2015. Nearly 700,000 people died from alcohol consumption in China, ranking the highest in the world in 2016. Pollutants known to be toxic to the liver include organic toxicants and heavy metals. Fried foods also contain a high amount of trans fats and toxic substances, both of which can injure the liver. Additionally, liver injuries can develop into a variety of illnesses, such as fatty liver, hepatitis, fibrosis, cirrhosis, and liver failure, as well as cancer [7]. Therefore, liver injury is regarded as a serious health problem, raising worldwide concern.

**Figure 1:**
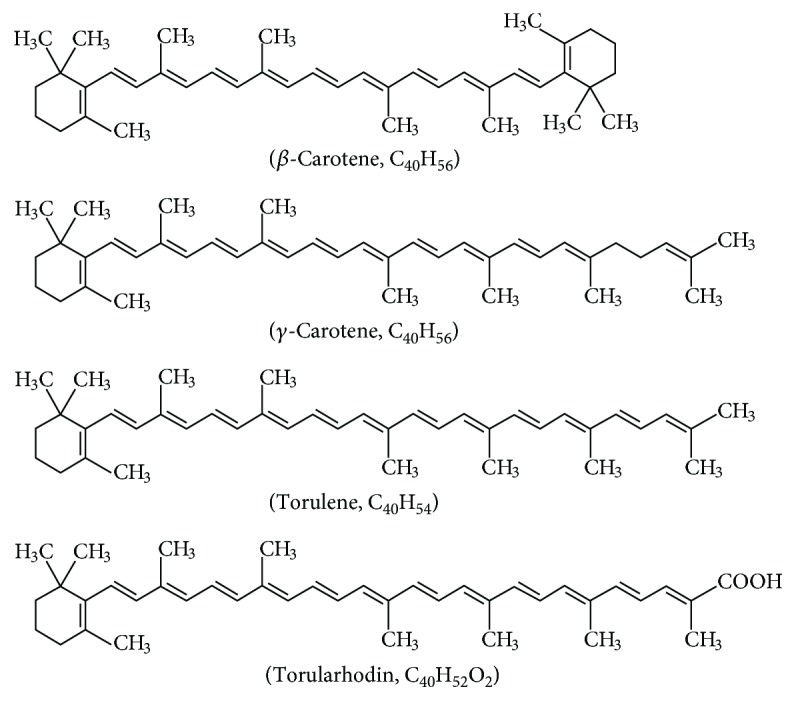
The chemical structures of the four carotenoids of *Sporidiobolus pararoseus.*

Carotenoids, which are organic compounds that belong to the family of 40-carbon terpenoids, occur naturally in fruits, vegetables, algae, fish, eggs, and oil [8, 9]. Until now, approximately 750 compounds of this type have been identified, out of which 50 compounds exhibit provitamin A activity [10–12]. They exert health-promoting effects, such as enhancing the immune system and accelerating wound healing, and can also be used to prevent organ injury owing to their antioxidative property [13, 14]. Humans are unable to biosynthesize carotenoids, and therefore, they must be supplied with diet [12]. Torularhodin is one of the most important carotenoids in *Sporidiobolus pararoseus* (Figure 1, including several other carotenoids in the yeast). Because it contains a hydroxyl group, it belongs to lutein. It has a noncyclic *β*-ionone ring and is the precursor of *β*-carotene [15]. Although torularhodin and lycopene are similar in structure, torularhodin has one more double bond [16–18]. Therefore, we surmised that the antioxidative activity of torularhodin was stronger than that of lycopene. Some research suggested that torularhodin from yeast has great scavenging activity toward peroxyl radicals and effectively inhibits degradation by singlet oxygen; thus, it increases cellular resistance to oxidation such as damage of cells induced by excessive selenium intake [6, 17, 19]. Other studies have indicated torularhodin as having antioxidative, anticancer, and antimicrobial activities [20–24], suggesting that it has good potential to protect the liver against oxidative damage.

The antioxidative function of torularhodin in liver injury has not been thoroughly studied. Therefore, the objective of the present study was to elucidate the antioxidative activity of torularhodin against oxidative damage in BRL cells. To elucidate the potential molecular mechanism underlying this process, injured BRL cells treated with torularhodin were used for transcriptome sequencing. Then, genes that were differentially expressed between the torularhodin-treated and control groups were identified, verified, and analyzed.

## 2. Materials and Methods

### 2.1. Materials

*Sporidiobolus pararoseus* JD-2 was obtained from the School of Biotechnology of Jiangnan University (China). Torularhodin was isolated and purified from the *S. pararoseus* extract according to a previously published method [25]. Its purity was greater than 95%, as determined by high-performance liquid chromatography with UV detection at 450 nm. Torularhodin was stored at -80°C; it was first dissolved in dimethyl sulfoxide (DMSO) and then in Dulbecco's modified Eagle's medium (DMEM) before use.

DMEM and fetal bovine serum (FBS) were from Gibco BRL (Grand Island, NY, USA). The DAPI dye and Cell Counting Kit-8 (CCK-8) were from Beyotime Biotechnology Co. Ltd. (Shanghai, China). The TRIzol Reagent was from Ambion (Kusatsu, Japan).

### 2.2. Cell Culture

BRL cells were obtained from the Cell Bank of the Chinese Academy of Sciences (Shanghai, China). The cells were incubated in DMEM containing 10% FBS under an atmosphere containing 5% CO_2_ at 37°C.

### 2.3. Cell Growth Assay

BRL cells were plated in 96-well plates at 5 × 10^3^ cells/well and incubated for 0, 12, 16, 24, 36, 48, and 60 h. After that, cell viability was analyzed with the CCK-8 reagents according to the manufacturer's instructions.

### 2.4. Determination of Different H_2_O_2_ Concentrations for Treatment

BRL cells were plated in 96-well plates at 5 × 10^3^ cells/well and incubated for 24 h. Then, H_2_O_2_ solutions at different concentrations (0, 100, 200, 300, 400, 500, 600, 700, 800, 900, and 1000 *μ*mol/ml) were added to the respective wells. After treatment for 8 h, cell viability was analyzed with the CCK-8 reagents according to the manufacturer's instructions.

### 2.5. Cell Viability Assay

BRL cells were plated in 96-well plates at 5 × 10^3^ cells/well and incubated for 24 h. Then, torularhodin solutions at different concentrations (0, 10^−7^, 10^−6^, 10^−5^, 10^−4^, 10^−3^, and 10^−2^ g/ml) were added to the respective wells (final DMSO concentration was less than 0.08%). After 16 h of torularhodin treatment, cell viability was analyzed with the CCK-8 reagents according to the manufacturer's instructions.

### 2.6. Morphological Observation and Determination of the Antioxidative Capacity of Torularhodin in BRL Cells

BRL cells in the logarithmic growth phase were seeded in 96-well plates at 5 × 10^3^ cells/well and incubated for 24 h. Then, the cells were divided into a normal control group (BRL cells were incubated for 24 h), an injury group (BRL cells were incubated for 16 h and then incubated with H_2_O_2_ for 8 h), and an intervention group (BRL cells were incubated with different concentrations of torularhodin solution (10^−7^, 10^−6^, 10^−5^, or 10^−4^ g/ml) for 16 h and then incubated with H_2_O_2_ for 8 h). Cell viability was analyzed with the CCK-8 reagents according to the manufacturer's instructions. Cell morphology images were obtained with microscope equipment (Leica Microsystems, Germany).

### 2.7. Immunofluorescence Staining

BRL cells were treated and incubated with H_2_O_2_ as described in Section 2.6. Then, the cells were fixed with 4% (*m*/*v*) paraformaldehyde, permeabilized with 0.1% Triton X-100, and blocked with 5% bovine serum albumin. Thereafter, the cells were stained with the primary antibody (anti-Superoxide Dismutase (SOD) rabbit polyclonal antibody and anti-COX IV mouse polyclonal antibody (Proteintech, USA)) and subsequently with Alexa Fluor 488-conjugated donkey anti-mouse secondary antibody or Alexa Fluor 568-conjugated donkey anti-rabbit secondary antibody (Invitrogen, USA). After cell staining, images were acquired with a confocal laser scanning microscope (Carl Zeis AG, Germany).

### 2.8. RNA Extraction and Analysis

BRL cells were treated and incubated with H_2_O_2_ as described in Section 2.5. Then, total RNA was extracted from the cells using TRIzol Reagent according to the manufacturer's instructions, and genomic DNA was removed using DNase I (TaKaRa, Dalian, China). Then, the RNA was sent to Majorbio (Shanghai, China) for sequencing. The RNA quality was determined with the 2100 Bioanalyzer (Agilent, Santa Clara, CA, USA) and quantified using the ND-2000 spectrophotometer (NanoDrop Technologies, Wilmington, DE, USA). Only high-quality RNA samples (concentration ≥ 200 ng/*μ*l, OD_260/280_ = 1.8 − 2.2) were used to construct the sequencing library. Then, the oligo(dT)-enriched mRNA was fragmented in a fragmentation buffer, following which the cleaved RNA fragments were reverse transcribed to establish the final cDNA library. After adaptor connection, paired-end sequencing was performed on an Illumina HiSeq 4000 System (Illumina, San Diego, CA, USA) according to the vendor's recommended protocol. Each group was tested with three biological replicates and three technical replicates. The pathways including statistically enriched genes were identified utilizing the Kyoto Encyclopedia of Genes and Genomes (KEGG, http://www.genome.jp/kegg) and Gene Ontology (GO, http://www.geneontology.org/) database analysis.

### 2.9. Statistical Analysis

All experimental data were from at least triple independent experiments. The results are presented as the means ± standard deviations (SD). One-way analysis of variance was conducted using data processing software. A *P* value of less than 0.05 was considered statistically significant.

## 3. Results

### 3.1. BRL Cell Growth Curve

**Figure 2:**
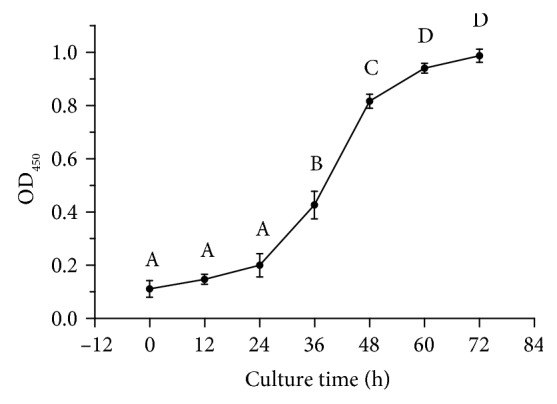
Growth curve of BRL cells. The values with different letters are significantly different (*P* < 0.05). All experiments were performed four times. Results are expressed as the means ± SD.

As shown in Figure 2, after 24 h of culture, the BRL cells entered the logarithmic growth phase. BRL cells at this stage were selected for subsequent experiments.

### 3.2. Effect of H_2_O_2_ on BRL Cell Viability

**Figure 3:**
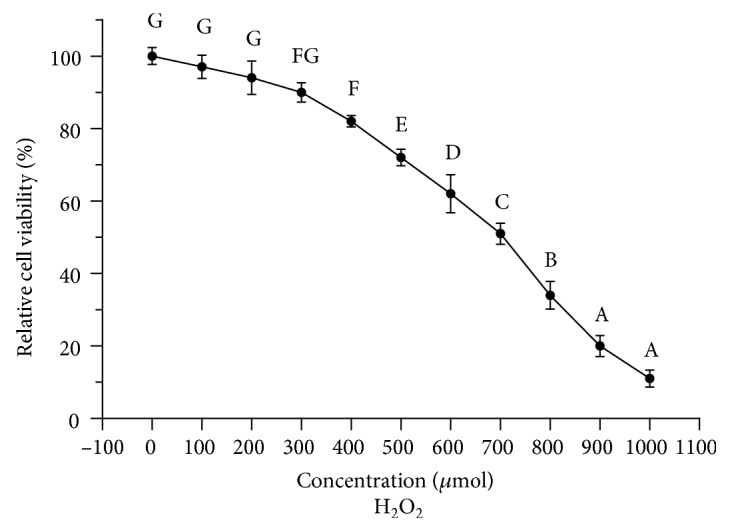
Effect of H_2_O_2_ on BRL cell viability. The values with different letters are significantly different (*P* < 0.05). All experiments were performed five times. Results are expressed as the means ± SD.

In order to determine the median lethal H_2_O_2_ concentration for BRL cells, the cells were incubated with different concentrations (0, 100, 200, 300, 400, 500, 600, 700, 800, 900, or 1000 *μ*mol/ml) of H_2_O_2_ for 8 h. As shown in Figure 3, the different concentrations of H_2_O_2_ had obvious inhibitory effects on cell proliferation, with the inhibitory action being dose dependent. At the 700 *μ*mol concentration of H_2_O_2_, the cell survival rate decreased to 51.26% (*P* < 0.05), which was the approximate half-lethal dose.

### 3.3. Effect of Torularhodin on BRL Cell Viability

**Figure 4:**
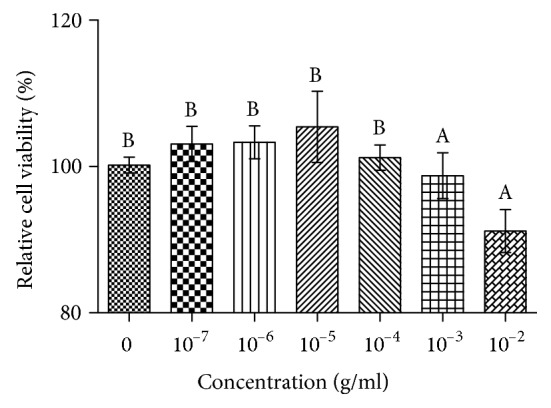
Effect of torularhodin on BRL cell viability. The values with different letters are significantly different (*P* < 0.05). All experiments were executed five times. Results are expressed as the means ± SD.

Before determining the antioxidative capacity of torularhodin in BRL cells, its toxicity toward this cell line needed to be tested. We used the CCK-8 assay to assess the effect of different concentrations of torularhodin on BRL cell viability. As shown in Figure 4, cell viability was not affected in the presence of torularhodin at less than or equal to 10^−4^ g/ml. However, cell viability was obviously inhibited when treated with torularhodin at 10^−3^ and 10^−2^ g/ml (*P* < 0.05). Therefore, a concentration of torularhodin less than or equal to 10^−4^ g/ml was chosen for the study.

### 3.4. Morphological Changes and Antioxidative Capacity of Torularhodin in BRL Cells

**Figure 5:**
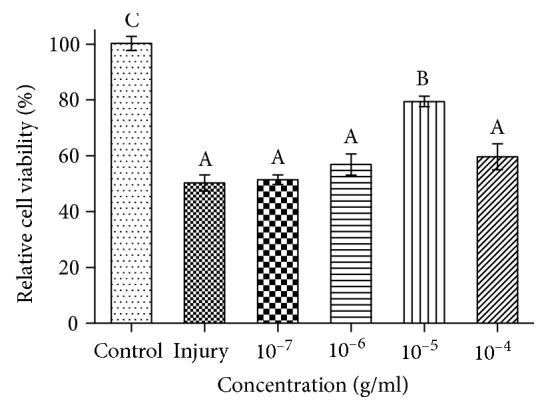
Effect of torularhodin on the viability of BRL cells with oxidative injury. The values with different letters are significantly different (*P* < 0.05). All experiments were executed five times. Results are expressed as the means ± SD.

Torularhodin at various concentrations (10^−7^, 10^−6^, 10^−5^, or 10^−4^ g/ml) was first incubated with BRL cells for 16 h, and then the cells were treated with H_2_O_2_ for 8 h. According to the cell viability assay (Figure 5), torularhodin at different concentrations had protective effects against cell damage by H_2_O_2_, particularly at 10^−5^ g/ml (*P* < 0.05).

**Figure 6:**
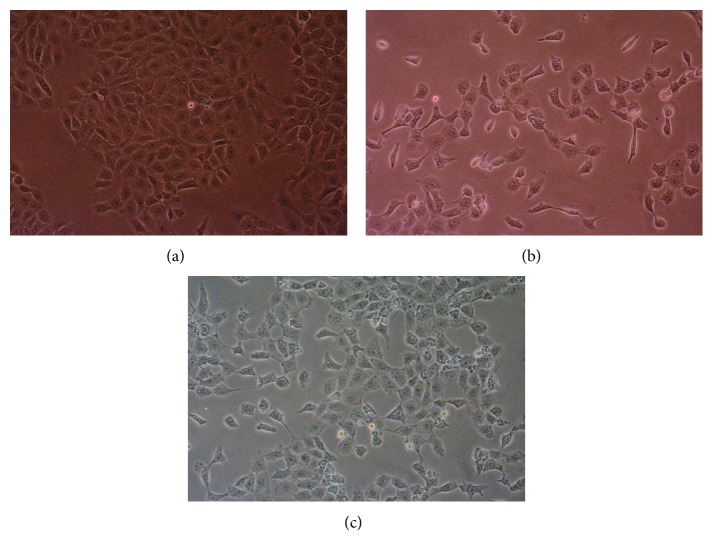
Protective effect of torularhodin on the morphology of BRL cells with oxidative injury. BRL cells were incubated for 24 h. Then, (a) BRL cells were incubated for 24 h. (b) BRL cells were incubated for 16 h and then incubated with H_2_O_2_ for 8 h. (c) BRL cells were incubated with different concentrations of torularhodin solution for 16 h and then incubated with H_2_O_2_ for 8 h. The cells were observed by microscopy (×200).

As shown in Figure 6(a), cells in the control group had a better growth status and appeared to be polygonal in shape with intact membrane integrity. The cells in the injury group were shrunken, and their number was obviously decreased (Figure 6(b)). Moreover, dead cells were observed in the medium. It was noteworthy that the oxidation-damaged cells treated with torularhodin were protected to a certain extent. As shown in Figure 6(c), most cells treated with torularhodin had unrestricted intercellular edges with intact membrane integrity, and their survival rate had increased significantly.

### 3.5. Immunofluorescence Observation

**Figure 7:**
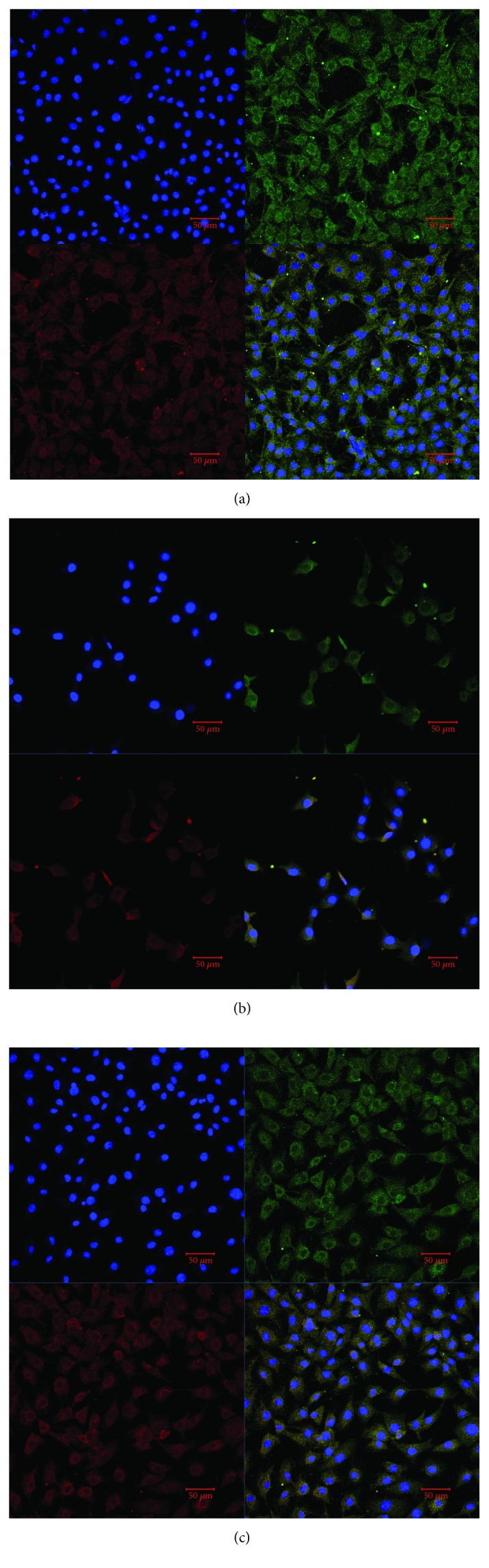
Immunofluorescence for the expression of related biomarkers in BRL cells. Cells were stained with superoxide dismutase (green), mitochondrion (red), and DAPI (blue). (a) Normal cultured cells. (b) H_2_O_2_ damages cells. (c) Cells treated with torularhodin. The protective effect of pigments on hydrogen peroxide-injured cells was remarkable. The pictures were captured at 40x magnification.

Figure 7 shows the immunofluorescence staining results for the control (Figure 7(a)), injury (Figure 7(b)), and intervention (Figure 7(c)) groups of cells. Compared with the control cells, the cells in the injury group had much fewer mitochondria and lower superoxide dismutase (SOD) activity. As expected, the number of cells in the injury group was the lowest. On the other hand, the intervention group had more cells, more mitochondria, and stronger SOD activity compared with the injury group. The results indicated that torularhodin could maintain cell integrity and enhance the antioxidative capacity in BRL cells.

### 3.6. Results of Transcriptome Analysis for the Torularhodin Intervention and Injury Groups

**Figure 8:**
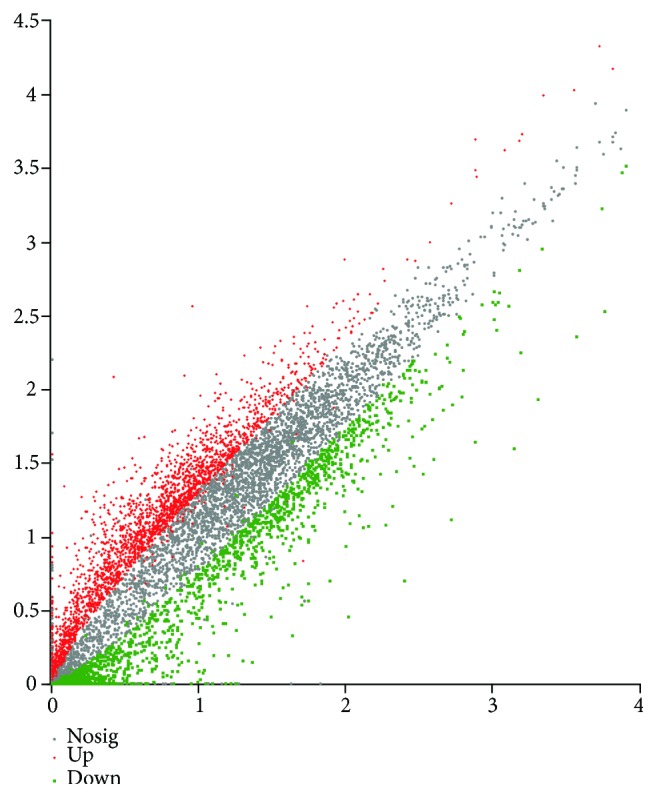
Scatter plot of differentially expressed genes between the torularhodin intervention and injury groups. The *x*-axis indicates the log2 expression values in the torularhodin intervention group, and the *y*-axis indicates the log2 expression values in the injury group. Each point indicates a particular gene or transcript. Red dots indicate upregulated genes, green dots indicate downregulated genes, and black dots indicate genes with a nonsignificant difference. After mapping all the genes, the nearer the point is to zero, the lower the expression level is, whereas the greater the degree of deviation is from the diagonal thread, the greater the difference in gene expression is between the two samples (*P* < 0.05).

The differentially expressed genes (DEGs) between the torularhodin intervention and injury groups were analyzed using the rat reference genome. The results (Figure 8) showed that a total of 2808 genes were significantly differentially expressed, with 1334 being upregulated and 1474 downregulated after torularhodin treatment.

**Figure 9:**
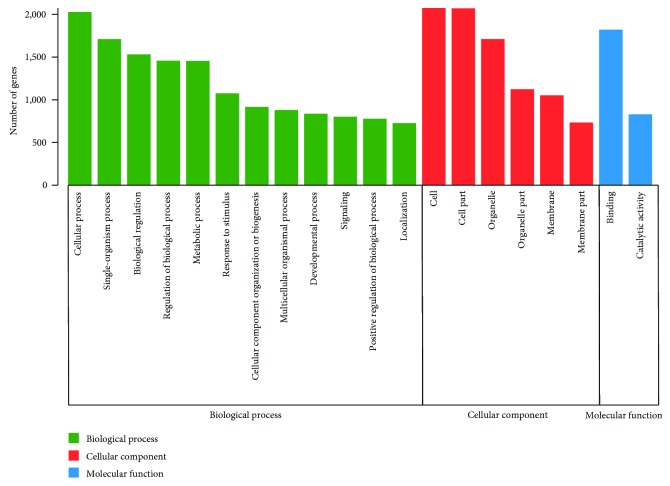
Histogram of the Gene Ontology annotation of the differentially expressed genes between the torularhodin intervention and injury groups. Green bars indicate genes annotated to biological process, red bars indicate genes annotated to cellular component, and blue bars indicate genes annotated to molecular function.

As shown in Figure 9, the DEGs between the torularhodin intervention and injury groups were classified by Gene Ontology (GO, http://www.geneontology.org/) enrichment into three main categories: biological process, cellular component, and molecular function. The most obvious difference found was in the biological process category. Thus, torularhodin could protect the biological process of cells under oxidative damage.

**Figure 10:**
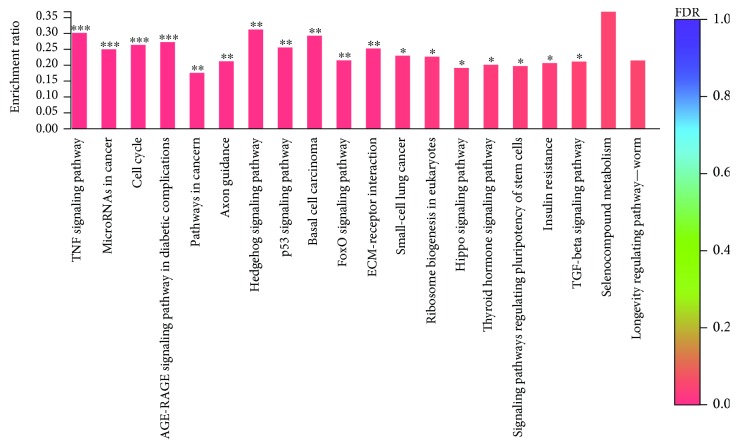
Histogram of the KEGG enrichment annotation of the differentially expressed genes between the torularhodin intervention and injury groups. FDR indicates the degree of KEGG enrichment; the darker the color, the more significant the effect of the KEGG gene. (∗ represents *P* < 0.05; ∗∗ represents *P* < 0.01; ∗∗∗ represents *P* < 0.001).

**Figure 11:**
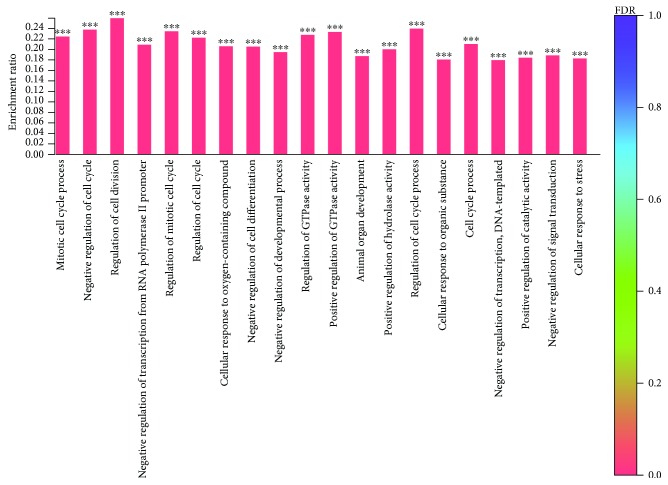
Histogram of the Gene Ontology (GO) enrichment annotation of the differentially expressed genes between the torularhodin intervention and injury groups. FDR indicates the degree of GO enrichment; the darker the color, the more significant the effect of the GO gene. (∗∗∗ represents *P* < 0.001).

According to the GO enrichment of the torularhodin-regulated DEGs, the main functions of these genes were in regulating cell cycle processes and enzymes in cells (Figure 10). Meanwhile, the results of the KEGG pathway enrichment analysis of the torularhodin-regulated DEGs showed that the main functions of these genes were related to cancer, antioxidation, and senility (Figure 11).

## 4. Discussion

Reactive oxygen species (ROS), which are oxidation products of cellular metabolism, can break DNA and oxidize proteins and lipids [26–28]. Usually, higher organisms can maintain a balance between oxidation and antioxidation. When organisms are exposed to harmful substances, they produce a stress reaction that breaks the balance between oxidation and antioxidation, leading to cell- and organism-level damage [29]. To some extent, the occurrence and aggravation of all diseases are directly or indirectly related to oxidative stress or the damage it causes [30]. The liver is susceptible to ROS-mediated damage because the ROS produced by the mitochondria, microsomes, and peroxisomes in parenchymal cells regulate peroxisome proliferator-activated receptor-alpha, which is mainly related to the expression of genes involved in liver fatty acid oxidation [28]. Excessive ROS accumulation disrupts the oxidative balance and leads to oxidative stress, which can cause or accelerate the occurrence of liver disease [31]. Oxidative stress can damage proteins, lipids, and DNA, and even change the pathways that control the normal physiological functions of organisms. Furthermore, the oxidative stress caused by liver disease can also cause injury to other organs of the body, such as kidney failure and brain impairment [32].

Carotenoids are a group of important natural pigments that are ubiquitous in animals, higher plants, fungi, and algae. They are the main source of vitamin A in vivo and also have antioxidative, immune regulatory, anticancer, and antiaging functions [11, 33]. Torularhodin is one such carotenoid. Studies have shown that torularhodin has a stronger ability than carotenes to scavenge peroxide free radicals [17]. Other studies suggested that torularhodin was more potent than *α*-tocopherol in inhibiting lipid peroxidation [34]. In the anticancer field, studies have shown that torularhodin has protective properties against the preneoplastic changes in the liver induced by dimethylnitrosamine and the ability to inhibit the development of prostate cancer [33, 35]. It is noteworthy that torularhodin also has a strong antioxidative capacity. Previous reports showed that torularhodin neutralized free radicals more efficiently than *β*-carotene [18]. Moreover, torularhodin also has strong antimicrobial activity [18]. Therefore, this carotenoid can protect organs by reducing the risks of oxidative stress, infection, and inflammation damage.

So far, research studies on torularhodin have mainly focused on their antioxidative, anticancer, and bacteriostatic effects, and there are few studies that have reported the molecular mechanism underlying its antioxidative activity. Our study found that at 10^−5^ g/ml, torularhodin had a significant protective effect against the oxidative damage of hepatocytes, with the results showing that cell viability and integrity were protected. This is considered due to the neutralization of free radicals by torularhodin, which can reduce the oxidative stress-induced cell damage and protect the integrity of cell membranes; thus, it maintains the normal morphology of cells and reduces their mortality. The immunofluorescence staining results indicated that torularhodin could maintain cell integrity and enhance the cellular antioxidative capacity. Torularhodin was considered to regulate the cell cycle and the activity of intracellular enzymes. The transcriptome analysis results showed that a total of 2808 genes were significantly differentially expressed. According to the GO enrichment analysis of the torularhodin-regulated DEGs, the main functions of these genes are in regulating cell cycle processes, enzymes in cells, and the cell response to oxygenated compounds. The response of cells to stress was similar to that in some previous studies, which was considered to be associated with the alleviation of cell damage [36, 37]. Therefore, torularhodin stabilizes the intracellular environment and increases cellular activity and thus increases the cell survival rate. Meanwhile, the KEGG pathway enrichment analysis showed that the main functions of these genes are relevant to cancer, antioxidation, cell cycle processes, metabolic pathways, and senility. This was similar to the GO results, in that the function of torularhodin was to protect cells from damage.

In addition, the transcriptome results showed that torularhodin could modulate the insulin metabolic pathway, likely because this pathway is affected by excessive ROS stimulation, in turn affecting other functions. As an antioxidant, torularhodin can improve this condition and protect the health of the cells and thus the organism as a whole. This was similar to other published research results [38]. The KEGG results also indicated that torularhodin modulated aging-related pathways and attenuated the effect of ROS on cells, thereby reducing the probability of premature cell aging [39]. We also found that torularhodin regulated the pathway involving p53, which is a tumor suppressor, and we speculate that the carotenoid attenuated oxidative stimulation to a certain extent, thereby reducing the likelihood of cancer [40]. In summary, torularhodin affects many cellular pathways, especially the anticancer and antioxidative pathways, and thus plays a significant role in stabilizing the intracellular environment.

In conclusion, we consider that torularhodin has a significant protective effect against the oxidative damage of hepatocytes; however, further study is necessary to verify this. The results showed that neutralization of free radicals, antioxidative and anticancer activities, and cell cycle pathways played an important role in this process of protection. The findings are similar to those of many studies on antioxidants. For example, Ungureanu and Ferdes showed the torularhodin had strong antioxidative activity, and Wu et al. concluded that torularhodin showed neuroprotective activity against H_2_O_2_-induced oxidative injury, related to its strong antioxidative activity [18, 20]. In the future, we will study the pathways and specific effects of carotenoids with the aim to utilize their full potential as antioxidants. To date, torularhodin has not been detected in foods. However, considering its obvious antioxidant capacity, torularhodin can be considered to be used as food additives and has a good commercial market prospect [12]. In addition, some studies suggested that torularhodin also had anticancer and antimicrobial activities. Du et al. confirmed that torularhodin at 18 mg/kg body mass significantly inhibited the development of prostate cancer in studied mice. Ungureanu and Ferdes also concluded that torularhodin showed antibacterial and antifungal properties toward all tested strains [18]. Therefore, we will research torularhodin further and explore its potential in the fields of medicine, health, and industrial production.

## Data Availability

The data used to support the findings of this study are available from the corresponding author upon request.
